# Trabectedin for Metastatic Soft Tissue Sarcoma: A Retrospective Single Center Analysis

**DOI:** 10.3390/md8102647

**Published:** 2010-10-13

**Authors:** Thomas Schmitt, Eva Keller, Sascha Dietrich, Patrick Wuchter, Anthony D. Ho, Gerlinde Egerer

**Affiliations:** Department of Internal Medicine V, Heidelberg University Clinics, Im Neuenheimer Feld 410, 69120 Heidelberg, Germany; E-Mails: Eva.Keller@med.uni-heidelberg.de (E.K.); Sascha.Dietrich@med.uni-heidelberg.de (S.D.); Patrick.Wuchter@med.uni-heidelberg.de (P.W.); Anthony.Ho@med.uni-heidelberg.de (A.D.H.); Gerlinde.Egerer@med.uni-heidelberg.de (G.E.)

**Keywords:** trabectedin, soft tissue sarcoma, metastatic, advanced, chemotherapy

## Abstract

Soft tissue sarcoma (STS) comprises a large variety of rare malignant tumors. Development of distant metastasis is frequent, even in patients undergoing initial curative surgery. Trabectedin, a tetrahydroisoquinoline alkaloid isolated from the Caribbean marine tunicate *Ecteinascidia turbinata*, was approved in 2007 for patients with advanced STS after failure of anthracyclines and ifosfamide, or for patients unsuited to receive these agents. In this study, we retrospectively analyzed 25 patients who had been treated with trabectedin at our institution between 2007 and 2010. The majority (72%) had been heavily pre-treated with ≥2 previous lines of chemotherapy. Response assessed by conventional RECIST criteria was low, with only one patient achieving a partial remission (PR) and 10 stable disease (SD) after three cycles of treatment. However, median progression-free survival (PFS) and overall survival (OS) were significantly prolonged in this population compared to non-responders, with 7.7 months *versus* 2.1 months (p < 0.0001; HR 15.37, 95% CI 4.3 to 54.5) and 12.13 months *versus* 5.54 months (p = 0.0137; HR 3.7, 95% CI 1.3 to 10.5), respectively. PFS for all patients was 58% at three months and 37% at six months. Side effects, including neutropenia, elevation of liver transaminases/liver function tests, and nausea/vomiting, were usually mild and manageable. However, dose reductions due to side effects were necessary in five patients. We conclude that trabectedin is an effective and generally well tolerated treatment for STS even in a heavily pre-treated patient population.

## 1. Introduction

Soft tissue sarcoma (STS) are a heterogeneous group of rare malignant tumors, predominantly arising from the embryonic mesoderm. For 2008, there were an estimated 10,390 new diagnoses and 3,680 deaths due to STS in the United States [1]. Because of treatment complexity, STS require a multidisciplinary approach in a specialized center involving oncologic surgeons, sarcoma pathologists, radiation therapists and medical oncologists. The mainstay of curative treatment is complete surgical resection with negative histological margins. However, development of distant metastases remains the major problem in up to 50% of all STS patients [2]. Prognosis for those individuals with advanced and unresectable disease is grim with a median survival of eight to 13 months from the start of first-line chemotherapy, usually doxorubicin and/or ifosfamid based regimens [3]. Response by conventional Response Evaluation Criteria in Solid Tumors (RECIST criteria) to those regimens is approximately 20–30% [4].

Trabectedin (Ecteinascidin-743 [ET-743], Yondelis®) is a tetrahydroisoquinoline alkaloid isolated from the Caribbean marine tunicate, *Ecteinascidia turbinata*, which binds through the minor groove of DNA at guanine nucleotides of specific sequences [5]. This new compound was approved in 2007 for second-line treatment of advanced STS in the European Union. Phase II studies identified 1.5 mg/m^2^ of trabectedin via 24-hour continuous intravenous (IV) infusion every three weeks as the optimal regimen. Overall response by RECIST criteria was low with only 4–17%. However, the six months progression free survival (PFS) was favorably comparable to other clinically used second-line treatments [6–8]. Interestingly, distinct histological subtypes seem to show major response rates to trabectedin, e.g., myxoid liposarcoma, expressing the chimeric FUS-CHOP fusion protein, and leiomyosarcoma [9,10]. Here we present a retrospective analysis of our experience treating STS patients with trabectedin.

## 2. Patients and Methods

### 2.1. Patients and Eligibility Criteria

All patients with metastatic STS treated at our institution between January 2007 and March 2010 were identified and retrospectively analyzed reviewing electronically filed patients’ charts. Eligibility criteria included histologically proven STS and failure of standard first-line doxorubicin-and/or ifosfamid-containing chemotherapy regimens. Tumor specimens were classified according to the Fédération Nationale des Centres de Lutte Contre le Cancer (FNCLCC) system. Patients with Ewing’s sarcoma or osteosarcoma were excluded from this analysis. Before start of treatment, and before continuing treatment with the subsequent cycle, the following criteria as defined by the manufacturer’s summary of product characteristics (SPC) were required: absolute neutrophile count (ANC) > 1.500/μL; platelets > 100,000/μL, bilirubin < upper limit of normal (ULN); alkaline phosphatase (AP) ≤ 2.5× ULN; alanine transaminase (ALT) and aspartate aminotransferase (AST) ≤ 2.5× ULN; creatinine clearance ≥ 30 mL/min, haemoglobin ≥ 9 g/dL, albumin > 25 g/L; creatine phosphokinase (CPK) ≤ 2.5 × ULN.

### 2.2. Trabectedin

After obtaining written informed consent, trabectedin was administered as a 1.5 mg/m2 24-hour continuous IV infusion every three weeks. Drug administration through an implantable port-catheter system or central venous line was mandatory by internal Standard Operating Procedures (SOPs). Premedication with dexamethasone 20 mg IV, 30 minutes prior to trabectedin, was mandatory. Antiemetic prophylaxis according to internal SOPs was administered with granisetron 2 mg po once daily on days 1 and 2. Treatment with trabectedin was discontinued at progression, in case of unacceptable toxicity or at the patient’s request.

### 2.3. Imaging Studies and Restaging

Assessment of tumor progression according to RECIST criteria [11] was performed after every 3 cycles of treatment or on finding clinical signs of progression by magnetic resonance imaging (MRI) and/or CT scans of primary tumor site and sites of metastases. Response to treatment was defined as complete remission (CR), partial remission (PR) or stable disease (SD).

### 2.4. Toxicity Assessment

Major clinical toxicities occurring after trabectedin were collected by chart review focusing on hematological toxicity, nausea/vomiting and changes in liver function tests. Dose limiting toxicities according to the manufacturer’s SPC: ANC < 500/μL for more than five days or neutropenia associated with fever/infection; thrombocytopenia < 25.000/μL; bilirubin > ULN; AP > 2.5× ULN; AST/ALT > 2.5× ULN. Dose reductions were performed if any of the above mentioned events occurred. Adverse events were graded according to Common Terminology Criteria for Adverse Events v4.0 (CTCAE), published May 28, 2009, by the National Cancer Institute (NCI). Patient charts were also reviewed for reports of drug extravasation including skin or soft tissue damage.

### 2.5. Statistical Analysis

Surviving patients were censored at the date of last follow up. Progression free survival (PFS) and overall survival (OS) were estimated using the method of Kaplan and Meier. PFS was defined as the time interval from the date of therapy induction to radiologically proven disease progression or patient’s death due to sarcoma related cause. Cause of death (COD) was determined by principal investigator (TS) by chart review and autopsy reports. OS was defined as the time interval from the date of therapy induction to patient’s death or last follow up. Statistical testing was done using the Logrank test. Significance levels were set at 0.05. Calculations were performed using Medcalc software (release 11; Mariakerke, Belgium). Data was analyzed as of May 04, 2010

## 3. Results and Discussion

### 3.1. Patient Characteristics

Between 2007 and 2010, 25 patients (male = 14, female = 11, median age 51 years [range 16–71]) with STS were treated at our institution. One heavily pre-treated patient with Ewing’s sarcoma received trabectedin resulting in disease progression after two cycles and was not included in this analysis. Nine patients (Pat. 016, 017, 018, 019, 021, 022, 023, 024, 025) were still alive at the date of data analysis with one subject (Pat. 023) still continuing treatment with trabectedin. Histological subtypes comprised leiomyosarcoma (n = 10), liposarcoma (n = 5), pleomorphic sarcoma (n = 3), synovial sarcoma (n = 3), rhabdomyosarcoma (n = 2) and other subtypes (n = 2) with tumor grades being G1 (n = 3), G2 (n = 4) and G3 (n = 18). No information on chromosomal aberrations or fusion products was available for any patient. Primary tumor sites included abdomen/retroperitoneum (n = 12), extremities (n = 9), trunk (n = 3), and head/neck (n = 1). Metastases were found in lung (n = 17), lymph nodes (n = 7), soft tissue/solid organs (n = 15) and bone (n = 4). Patients had received 1 (n = 7), 2 (n = 10) or ≥3 (n = 8) previous lines of chemotherapy. A total number of 103 cycles were administered with a median number of four cycles (range 1–12) received by each patient. No patient underwent further surgery after administration of trabectedin. Patient characteristics are summarized in Table 1.

### 3.2. Response Assessment

**Figure 1:**
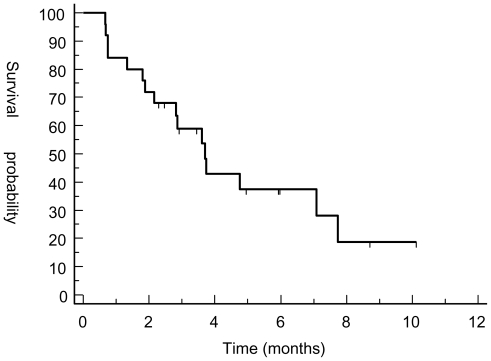
Progression-free survival for all patients. Progression-free survival (months) from start of trabectedin to disease progression was estimated using the method of Kaplan and Meier.

**Figure 2:**
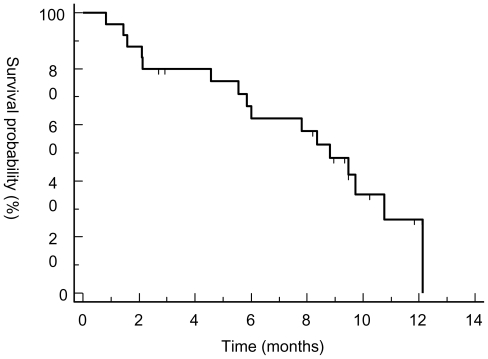
Overall survival for all patients. Overall survival from the start of trabectedin to disease progression (months) was estimated using the method of Kaplan and Meier.

**Figure 3:**
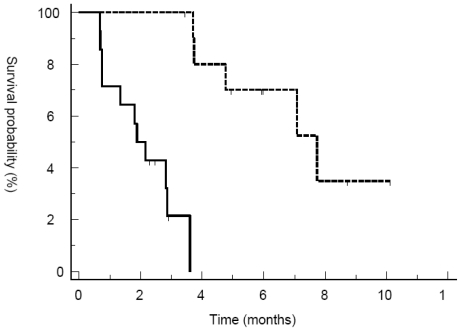
Progression-free survival in responders *vs.* non-responders. Progression-free survival (months) from start of trabectedin to disease progression was estimated using the method of Kaplan and Meier. Responders (dotted line), defined as CR, PR or SD after three cycles of treatment, showed significantly prolonged PFS compared to non-responders (solid line).

**Figure 4:**
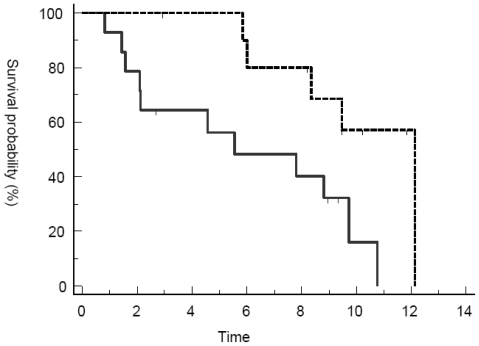
Overall survival in responders *vs.* non-responders. Overall survival (months) from the start of trabectedin to death was estimated using the method of Kaplan and Meier. Responders (dotted line), defined as CR, PR or SD after three cycles of treatment, showed significantly prolonged OS compared to non-responders (solid line).

Best response by RECIST criteria after three cycles of treatment were PR (n = 1) and SD (n = 10). Primarily progressive disease was seen in 14 patients. Two patients (Pat. 017 and UPN 018) discontinued treatment after six and 12 cycles of therapy achieving SD. Median PFS and OS for the whole population were 3.7 and 8.8 months, respectively. PFS at three and six months was 58% and 37% (Figures 1 and 2). However, patients responding to therapy (defined as PR or SD after three cycles of treatment with trabectedin) had a significantly longer median PFS and OS with 7.7 months *versus* 2.1 months (p < 0.0001; HR 15.37, 95% CI 4.3 to 54.5) and 12.13 months *versus* 5.54 months (p = 0.0137; HR 3.7, 95% CI 1.3 to 10.5) (Figure 3 and 4). In general, response assessment by different histological subsets was not possible due to small patient numbers. However, comparing patients with leiomyosarcoma *versus* other histological subtypes, we found a significant difference in median OS (10.8 *vs.* 7.8 months; p = 0.01; HR 4.5, 95% CI 1.4 to 14.4) but not in PFS (3.7 *vs.* 2.9 months;

p = 0.14). Comparing PFS and OS in G1 *versus* G2/3 tumors and 1–2 *versus* ≥3 previous lines of chemotherapy, respectively, we found no statistically significant differences.

### 3.3. Toxicity Assessment

Major toxicities included nausea/vomiting CTC grade ≥ 2 (n = 4), neutropenia (n = 4) and elevation of liver transaminases > 2.5× ULN (n = 2), leading to dose reductions in five patients. One patient who developed elevation of liver transaminases had concomitant radiological disease progression and therefore did not receive further treatment with trabectedin. Skin or soft tissue damage due to drug extravasation was not observed in any patient. No patient discontinued treatment due to unacceptable toxicities.

### 3.4. Discussion

Treatment of metastatic STS is challenging and outcome is frequently grim. Therefore, new therapeutic targets and compounds are urgently needed. Trabectedin was approved in 2007 for patients with advanced STS after failure of anthracyclines and ifosfamide, or those who were unsuited to receive these agents. In this study we retrospectively analyzed 25 patients treated at our institution. Only seven out of 25 patients (28%) received trabectedin as second-line therapy. The majority (72%) had been heavily pre-treated with ≥2 previous lines of chemotherapy. Conventional first-line chemotherapy with doxorubicin and/or ifosfamide containing regimens results in radiological response rates of approximately 20–30%. However, there has been an ongoing debate whether RECIST criteria reflect the specific biology of STS [12,13]. Van Glabbeke *et al.* suggested already in 2002 to use PFS rates at six and three months instead of tumor response by RECIST criteria to assess drug activity in clinical trials [14]. Based on the success of functional imaging in gastrointestinal stroma tumors (GIST), approaches with fluorine-18-fluorodeoxyglucose PET (FDG-PET) and diffusion weighted MRI scans [15–17] have been explored in STS to assess treatment response. Data on the usefulness of functional imaging in patients treated with trabectedin is scarce. Our own group reported on nine patients monitored with FDG-PET [18]. In this small series, stabilization of standardized uptake values (SUV) was observed in nearly all patients. One patient with morphologic PR showed a 40% decrease in SUV. However, further studies are needed to validate those results.

As seen in previous studies with trabectedin, our current series confirms that radiological response by RECIST criteria is low. Only one patient (4%) achieved PR after three cycles of treatment. However, tumor control was obtained in a further 10 patients (40%) achieving SD, leading to a median PFS of 7.7 months and median OS of 12.13 months in this group. PFS at six months for the whole population was 37% and median PFS 3.7 months. Statistical testing might be slightly influenced by the fact that at the date of data analysis nine patients were still in follow up. However, our results are comparable to other clinically used second-line treatments for metastatic STS and the previously published phase II studies with trabectedin. Here, PFS rates of 24–29% at six months and a median PFS of 1.7–3.4 months, respectively, were reported [6–8]. Further reports of trabectedin in routine use confirm our results [19,20].

Other commonly used second-line regimens include gemcitabine or gemcitabine plus docetaxel. However, these agents have not been officially approved for the treatment of metastatic STS. For gemcitabine monotherapy, a PFS rate of 13.3% has been reported at six months [21], which is slightly below the suggested 14% to assess drug activity at this time point as reported by Van Glabbeke *et al*. [14]. Furthermore, the additional effect of docetaxel has been questioned. Maki *et al*. reported a median PFS of 6.2 months for the combination regimen and 3.0 months for gemcitabine monotherapy [22]. In contrast, Duffaud *et al*. concluded that there was no benefit for the combination regimen as median PFS was 5.5 months for gemcitabine alone *versus* 3.4 months for gemcitabine plus docetaxel [23]. However, response may differ in histological subsets.

Trabectedin does not act exclusively as a minor groove DNA binder, but has additional effects at the transcriptional level [24]. Exploiting these additional molecular properties of trabectedin, combination regimens with doxorubicin have been tested with preliminary promising results [25,26]. Furthermore, this might explain its particular activity in myxoid liposarcoma (MLS) characterized by the translocations t(12;16)(q13;p11) or t(12;22)(q13;q12) resulting in the formation of FUS-CHOP and EWS-CHOP fusion proteins, respectively. A superior overall response rate of 51% with a median PFS of 14 months and a PFS rate of 88% at six months was demonstrated [9]. *In vivo*, maturation of MLS lipoblasts after treatment with trabectedin has been shown, elucidating the underlying pathophysiological pathways [27]. Unfortunately, patient numbers in our series were too small to statistically assess response by different histological subtypes. One subject (Pat. 009) with MLS showed disease progression after five cycles of treatment. For patients with leiomyosarcoma, we found no statistically significant difference in median PFS. However, this subgroup showed improved median OS. In previous studies, distinct susceptibility to trabectedin in this histological subtype was suggested [10]. Most probably due to the small patient number, we were not able to reproduce these results.

The rate of major toxicities and adverse events was low in our retrospective analysis and in line with the manufacturer’s SPC. As administration through a port-catheter system or central venous line was mandatory due to internal SOPs, no patient experienced drug extravasation. However, there have been reports of skin and soft tissue damage [28].

## 4. Conclusion

Trabectedin is a generally well tolerated and effective treatment for STS even in a heavily pre-treated patient population. Major toxicities include neutropenia, changes in liver function tests and nausea/vomiting. Dose reductions usually allow continuation of treatment. Response by conventional RECIST criteria is low. However, progression-free survival and overall survival were significantly prolonged in responders and seem not to be inferior to other clinically used second-line treatments for the whole patient population.

## Figures and Tables

**Table 1:** Patient characteristics.

Patient	Age	Gender	Histology	Grading	Primary tumor	Metastases	Previous CTX lines	#Cycles	Staging	PFS (in days)	OS (in days)	Complications	Alive	COD
001	71	M	Spindle cell sarcoma	G3	Trunk	S	2	2	PD	21	64	None	No	Progression
002	64	M	Leiomyosarcoma	G3	Extremity	P	2	2	PD	23	48	None	No	Progression
003	36	F	Liposarcoma	G2	Abdomen	S, P	2	3	SD	145	255	None	No	Progression
004	16	F	Synovial sarcoma	G3	Trunk	S, P	6	1	PD	23	44	None	No	Progression
005	53	F	Liposarcoma	G3	Retroperitoneum	S	3	3	PR	236	289	None	No	Progression
006	55	M	Pleomorphic Sarcoma	G3	Extremity	P, S	3	3	PD	87	139	None	No	Progression
007	56	M	Pleomorphic Sarcoma	G3	Abdomen	P	2	5	PD	110	269	None	No	Progression
008	29	F	Leiomyosarcoma	G3	Abdomen	S, P	4	3	PD	86	169	N/V, liver tox	No	Progression
009	46	M	Myxoid Liposarcoma	G1	Extremity	S, B	2	5	SD	114	183	None	No	Progression
010	56	F	Leiomyosarcoma	G3	Abdomen	S, P	2	7	SD	216	370	None	No	Progression
011	58	M	Liposarcoma	G3	Retroperitoneum	S	3	3	PD	55	238	None	No	Progression
012	37	M	Leiomyosarcoma	G3	Abdomen	S, P, L	1	6	SD	113	178	None	No	Progression
013	57	M	Synovial sarcoma	G3	Extremity	P, L	3	1	PD	20	25	None	No	Progression
014	70	F	Leiomyosarcoma	G3	Abdomen	P	2	3	PD	66	297	N/V	No	Progression
015	49	F	Leiomyosarcoma	G2	Abdomen	S, P, L	1	2	PD	57	328	N/V	No	Progression
016	49	F	Leiomyosarcoma	G1	Abdomen	P	1	8	SD	182	240	hem tox	Yes	
017	64	M	Leiomyosarcoma	G2	Extremity	P, B	4	6	SD	309	309	hem tox	Yes	
018	49	F	Leiomyosarcoma	G3	Abdomen	S	2	12	SD	266	266	None	Yes	
019	53	M	Leiomyosarcoma	G2	Extremity	S, B	2	3	PD	75	237	hem tox	Yes	
020	49	M	Synovial sarcoma	G3	Trunk	P, L	1	1	PD	41	65	liver tox	No	Progression
021	31	F	Pleomorphic Sarcoma	G3	Abdomen	L, B	2	3	PD	70	212	N/V	Yes	
022	71	M	Liposarcoma	G3	Extremity	S, P	3	8	SD	181	205	hem tox	Yes	
023	58	M	Rhabdomyosarcoma	G3	Head/Neck	L	1	5	SD	105	105	None	Yes	
024	24	F	Fibromyxoid sarcoma	G1	Extremity	S, P	1	5	SD	151	188	None	Yes	
025	64	M	Rhabdomyosarcoma	G3	Extremity	P, L	1	3	PD	89	89	None	Yes	

**Abbreviations**: B = bone metastasis; Dx = diagnosis; COD = cause of death; F = female; Hem tox = hematological toxicity; Liver tox = liver toxicity; M = male; N/V = nausea/vomiting; OS = overall survival; P = pulmonary metastasis; PD = progressive disease; PFS = progression-free survival; PR = partial remission; S = soft tissue/solid organ metastasis; SD = stable disease.
